# Superior protection in a relapsing *Plasmodium cynomolgi* rhesus macaque model by a chemoprophylaxis with sporozoite immunization regimen with atovaquone-proguanil followed by primaquine

**DOI:** 10.1186/s12936-024-04933-y

**Published:** 2024-04-17

**Authors:** Kosol Yongvanitchit, Utaiwan Kum-Arb, Amporn Limsalakpetch, Rawiwan Im-Erbsin, Ratawan Ubalee, Michele D. Spring, Brian A. Vesely, Norman Waters, Sathit Pichyangkul

**Affiliations:** Armed Forces Research Institute of Medical Sciences (AFRIMS), Bangkok, Thailand

**Keywords:** Relapsing *Plasmodium cynomolgi* rhesus macaque model, Chemoprophylaxis with sporozoite immunization, Atovaquone-proguanil, Sporozoite-specific T cell responses

## Abstract

**Background:**

To gain a deeper understanding of protective immunity against relapsing malaria, this study examined sporozoite-specific T cell responses induced by a chemoprophylaxis with sporozoite (CPS) immunization in a relapsing *Plasmodium cynomolgi* rhesus macaque model.

**Methods:**

The animals received three CPS immunizations with *P. cynomolgi* sporozoites, administered by mosquito bite, while under two anti-malarial drug regimens. Group 1 (n = 6) received artesunate/chloroquine (AS/CQ) followed by a radical cure with CQ plus primaquine (PQ). Group 2 (n = 6) received atovaquone-proguanil (AP) followed by PQ. After the final immunization, the animals were challenged with intravenous injection of 10^4^ *P. cynomolgi* sporozoites, the dose that induced reliable infection and relapse rate. These animals, along with control animals (n = 6), were monitored for primary infection and subsequent relapses. Immunogenicity blood draws were done after each of the three CPS session, before and after the challenge, with liver, spleen and bone marrow sampling and analysis done after the challenge.

**Results:**

Group 2 animals demonstrated superior protection, with two achieving protection and two experiencing partial protection, while only one animal in group 1 had partial protection. These animals displayed high sporozoite-specific IFN-γ T cell responses in the liver, spleen, and bone marrow after the challenge with one protected animal having the highest frequency of IFN-γ^+^ CD8^+^, IFN-γ^+^ CD4^+^, and IFN-γ^+^ γδ T cells in the liver. Partially protected animals also demonstrated a relatively high frequency of IFN-γ^+^ CD8^+^, IFN-γ^+^ CD4^+^, and IFN-γ^+^ γδ T cells in the liver. It is important to highlight that the second animal in group 2, which experienced protection, exhibited deficient sporozoite-specific T cell responses in the liver while displaying average to high T cell responses in the spleen and bone marrow.

**Conclusions:**

This research supports the notion that local liver T cell immunity plays a crucial role in defending against liver-stage infection. Nevertheless, there is an instance where protection occurs independently of T cell responses in the liver, suggesting the involvement of the liver's innate immunity. The relapsing *P. cynomolgi* rhesus macaque model holds promise for informing the development of vaccines against relapsing *P. vivax*.

**Supplementary Information:**

The online version contains supplementary material available at 10.1186/s12936-024-04933-y.

## Background

In 2022, there were approximately 249 million malaria cases and 608,000 deaths worldwide. The African region accounted for the majority, with 94% of cases and 95% of deaths, and children under 5 represented 80% of fatalities in the region [1]. While *Plasmodium falciparum* causes the most significant morbidity and mortality, *Plasmodium vivax* is widely geographically distributed and can lead to severe disease [2]. *Plasmodium vivax* infection presents critical, unsolved treatment challenges due to the presence of dormant liver stages (hypnozoites) that can lead to frequent relapses [3–5]. These relapses pose a significant burden on affected individuals and complicate malaria control efforts.

Current treatment options, such as primaquine and tafenoquine, are limited by serious side effects, including acute hemolysis in individuals with glucose-6-phosphate dehydrogenase (G6PD) deficiency [6, 7]. Additionally, these drugs cannot be administered during pregnancy or to young children under the age of 6 months [8]. Therefore, the development of a safe and effective vaccine that can prevent *P. vivax* infection and subsequent relapses is crucial for accelerating its elimination.

While efforts have been made to develop a vaccine against *P. vivax*, no licensed vaccine exists to date. The single vaccine candidate against *P. vivax* circumsporozoite protein (CSP) tested in the US, VMP001/AS01B, was found to be safe and immunogenic but conferred no sterile protection [9]. All study subjects were then treated with a radical cure course of primaquine, therefore, residual efficacy against relapse was not determined. A recent phase II trial of the PvCS vaccine, using three long synthetic peptides (LSP) covering the amino-terminal (N), central repeats (R), and C-terminal regions of CSP formulated with the adjuvant Montanide ISA-51, was found to be safe and significantly protected both naïve and semi-immune volunteers [10]. Further studies are being conducted with a larger number of participants to confirm the results (NCT 04739917).

To design effective vaccines that can prevent *P. vivax* infection and elimination, it is necessary to elucidate potential protective immune responses against both the primary infection and relapse. Employing relevant biological animal models, such as *Plasmodium cynomolgi*, a relapsing nonhuman primate malaria parasite, in rhesus macaques (*Macaca mulatta*) [11], may accelerate testing and down-selection of *P. vivax* vaccine candidates.

Chemoprophylaxis with sporozoite (CPS) immunization is an approach to induce a protective immune response by inoculating sporozoites under antimalarial drugs [12]. Multiple recent studies have shown that *P. falciparum* CPS immunization has provided consistent and effective protection in both animals and humans [12–15]. In a previous study using *P. knowlesi* CPS under CQ in rhesus monkeys, the regimen provided sterile protection against sporozoite challenge in 50% of immunized animals and induced high levels of liver-resident sporozoite-specific memory T cell responses [16]. This finding underscores the significant role played by liver tissue-resident memory T cells in protection against malaria liver-stage infection. It is worth noting that an innate response of the liver could also contribute to control malaria liver-stage infections. A recent investigation has showed that an effective type I IFN response in the infected hepatocytes can control malaria liver-stage infection [17].

So far, CPS immunization has not been used to evaluate sporozoite-specific T cell responses against relapsing *P. cynomolgi* in rhesus macaques. In this study, CPS immunization approaches with two drug regimens were assessed using a relapsing *P. cynomolgi* rhesus macaque model: (1) drug that affects blood-stage parasites but not liver-stage parasites (AS or CQ), and (2) drug that affects both blood-stage parasites and liver stage schizonts (AP). The former would provide evidence that immune responses were mainly effective against both liver stage schizonts and hypnozoites. This would enable comparisons with the second regimen, which eradicated both blood-stage parasites and the liver stage schizonts. Consequently, the immune responses can be attributed specifically to hypnozoites. Each regimen underwent efficacy testing and evaluations for multi-compartment T cell responses.

## Methods

### Ethics approval

The animals were housed and looked after at a facility that had received accreditation from the Association for Assessment and Accreditation of Laboratory Animal Care, International (AAALAC). Their care was in adherence to the Animal Welfare Act and the eighth edition of the Guide for the Care and Use of Laboratory Animals (National Research Council, 2011), as well as all relevant guidelines from the USDA, Office of Laboratory Animal Welfare, and Department of Defense. All animal procedures were approved by The United States Army Medical Directorate-Armed Forces Research Medical Sciences (USAMD-AFRIMS) Institutional Animal Care and Use Committee (IACUC). Rhesus macaques used throughout the study were of Indian origin (both male and female), with a weight range of 5–10 kg, and an age range of 3–10 years. The animals were malaria-naïve and had no prior exposure to any malaria parasites, including both *P. knowlesi* and *P. cynomolgi*. The researchers were not blinded to the study groups.

### Preparation of *P. cynomolgi* sporozoites

*Anopheles dirus* mosquitoes were maintained in the insectary of the Entomology Department, AFRIMS, Bangkok, Thailand*. Plasmodium cynomolgi* (B strain)-infected mosquitoes were prepared as previously described [11]. Briefly, a total of 500 to 1000 *Plasmodium* naïve mosquitoes were fed on the anesthetized infected-donor monkey. The gametocytes required 14–16 days to complete the sporogony in mosquitoes, and sporozoites were harvested from the mosquitoes’ salivary glands.

### Determination of a *Plasmodium cynomolgi* sporozoite challenge dose for a vaccine study

The animals were administered intravenous (IV) injections of sporozoites at varying concentrations of 10^6^, 10^5^, 10^4^, and 10^3^ sporozoites in 1 ml of phosphate buffered saline (PBS), (Sigma-Aldrich, St. Louis, MO) containing 5% bovine serum albumin (BSA) (Sigma-Aldrich). To serve as a positive control, a dose of 10^6^ sporozoites was used for comparison. Parasitaemia was determined using both thick blood smears (low parasitaemia) and thin blood smears (high parasitaemia). Once the parasitaemia level reached 5000/µl after each episode, the animals were treated with chloroquine (CQ) (TOKYO Chemical Industry, Tokyo, Japan) orally at a dosage of 10 mg/kg/day for 7 days. After the second relapse, the animals were orally administered a radical cure of chloroquine (CQ) at a dosage of 10 mg/kg/day for 7 days, along with primaquine (PQ) (Walter Reed Army Institute of Research (WRAIR), Silver Spring, MD) at a dosage of 1.78 mg/kg/day for 7 days. A low sporozoite dose, consistently inducing primary infection and two relapses, was then employed to challenge animals in the CPS immunization study.

### Animal randomization and sample size for CPS immunization study

This is a proof-of-concept study assessing the protective efficacy of CPS immunization in a relapsing *P. cynomolgi* rhesus macaque model. In the vaccine efficacy experiment, healthy animals were randomly divided into three groups, each consisting of six animals, ensuring a balanced distribution across treatments (nQuery Advisor 7.0). Two groups received CPS vaccines, and one group served as a control.

When there are 6 animals in each group (vaccine and control group), totaling 4 animals with no protection, and using a 0.05 level one-sided log-rank test to compare survival curves based on data from a previous study [16] indicating protection rates of 0.5 and 0.0001 for the vaccine group and control group, respectively, there would be 82% power to detect the differences between the two groups. This calculation assumes no dropouts prior to study completion [18, 19].

### CPS immunization and challenge

**Fig. 1:**
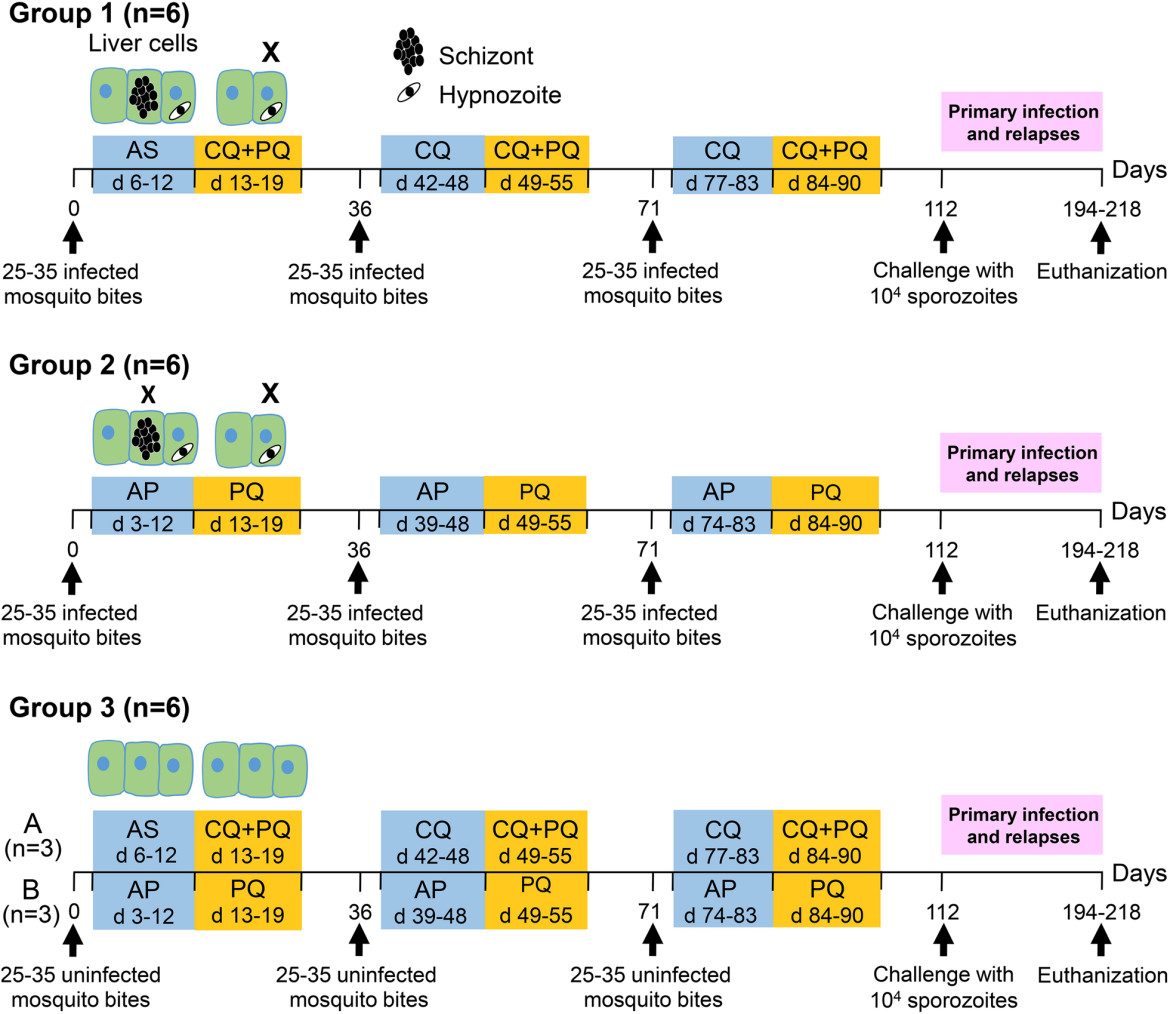
The study design flowchart depicts the randomization of 18 healthy rhesus monkeys into three groups. Group 1 (n = 6) and group 2 (n = 6) animals received three CPS immunizations with 25–35 bites of *P. cynomolgi*-infected mosquitoes under two different drug regimens. Control animals in group 3 (n = 6) received 25–35 bites from uninfected mosquitoes under either the same drug regimen as animals in group 1 (**A**; n = 3) or as group 2 (**B**; n = 3). After the final immunization, all animals were challenged with an IV injection of 10^4^ *P. cynomolgi* sporozoites on day 112 and then monitored for primary infection and subsequent relapses

In this experiment, 18 healthy animals were divided into 3 groups (Fig. 1). Group 1 (n = 6) animals received 3 sequences of bites from 25 to 35 *P. cynomolgi*-infected mosquitoes on the middle of the abdomen at days 0, 36, and 71. Salivary glands from all blood-engorged mosquitoes were removed and inspected to verify the existence of sporozoites. Mosquito feeding was performed repeatedly, if required, until each animal had received a minimum of 25 bites and a maximum of 35 bites from infected mosquitoes. During the first immunization, animals in this group received treatment with intravenous injection of AS (Guilin Pharmaceutical, Shanghai, China) (4 mg/kg/day for 7 days) to kill blood-stage parasites; however, low parasitaemia levels were still detected in one animal after the 7-day treatment (Additional file 1: Fig. S1). Therefore, a radical cure was administered consisting of CQ plus PQ to all animals in this group. For the second and third immunization, the drug regimens in group 1 animals were changed to CQ (7 days) and then followed by a radical cure of CQ plus PQ treatment (7 days) to ensure blood-stage parasite killing and hypnozoite elimination to prevent relapse [11]. This group was called AS/CQ – CQ + PQ.

Group 2 animals (n = 6) were subjected to similar sporozoite immunization as group 1, except they were orally administered with AP (WRAIR) at 15–6 mg/kg/day for 10 days to eliminate both blood-stage parasites and liver stage schizonts. The dose of AP was calculated from the human dose after application of a scaling factor to adjust for body surface area (Geoffrey Dow, unpublished data). Subsequently, PQ was given for 7 days to remove hypnozoites and this group was named AP – PQ. Meanwhile, group 3 animals (n = 6) were exposed to 25–35 bites from uninfected mosquitoes and were either subjected to the same drug regimen as group 1 (A; n = 3) or group 2 (B; n = 3) animals, serving as control.

At day 112, all animals were challenged with IV injection of 10^4^ *P. cynomolgi* sporozoites. Upon reaching a parasitaemia level of 5000 parasites/µl of blood, each episode of parasitaemia was treated with CQ for 7 days. The animals were monitored until days 194–218 post sporozoite challenge to assess primary infection and subsequent relapses. Protection was defined as the absence of blood-stage parasite detection. This was determined by thin and thick blood smear readings following the challenge.

### Euthanasia

All animals were humanely euthanized at the end of the study. Briefly, the animals were chemically restrained with an intramuscular injection of ketamine hydrochloride (Covetrus North America, Dublin, OH), (20 mg/kg). After sedation, an injectable commercial euthanasia agent (Fatal-Plus) from Vortech Pharmaceuticals (Dearborn, MI) was administered intravenously at a dose of 86 mg/kg of sodium pentobarbital to induce euthanasia. Animals were euthanized at different time points based on the time of the last detected parasitaemia. Due to the time constraint, only one animal could be euthanized per day for the harvesting of tissues and isolation of immune cells.

### Preparation of PBMCs and mononuclear immune cells

Peripheral blood was collected at baseline, 14 days after each immunization, prior to challenge, and post-challenge, and then processed for PBMC isolation by centrifugation using Histopaque-1077 (Sigma-Aldrich). The liver, spleen, and bone marrow were collected immediately after euthanasia. The spleen and bone marrow were homogenized between the frosted ends of two slides, while the liver tissues were cut into small pieces and homogenized in the presence of 1 μg/ml of collagenase type 1 (Life Technologies, Grand Island, NY) using automated tissue dissociation (Miltenyi Biotec, Auburn, CA). The samples were then incubated at 37 °C for another 1 h. Digested liver cells, as well as cells derived from the spleen and bone marrow, were passed through 70 μm cell strainers (BD Falcon, Durham NC). Subsequently, single cell suspensions were obtained by centrifugation using Histopaque-1077 to isolate mononuclear immune cells. Contaminated red blood cells were removed by treating the cells with an ammonium chloride-based lysing solution (BD Biosciences, San Jose, CA). PBMCs and mononuclear immune cells derived from different tissues were suspended in cold freezing media consisting of 10% dimethyl sulfoxide (DMSO) (Sigma-Aldrich) and 90% heat inactivated fetal bovine serum (FBS) (Gibco, Grand Island, NY), with a cell concentration of 10 × 10^6^ cells/ml/vial. Vials were kept in freezing container (Mr. Frosty, Nalgene, Rochester, NY) and placed in a − 80 °C freezer overnight before being stored in liquid nitrogen until use.

### Antibody response

The immunofluorescence assay (IFA) was used to assess antibody responses against whole sporozoites and blood-stage schizonts. To prepare sporozoite slides, separate multiwell slides (Electron Microscopy Sciences, Hatfield, PA) were coated with *P. cynomolgi* sporozoites (10^4^/well), air dried, and fixed with methanol. For blood-stage schizont slides, *P. cynomolgi-*infected red blood cells were cultured in McCoy's 5A Medium (Gibco) plus sodium bicarbonate and 20% heat inactivated monkey serum overnight in 5% CO_2_ incubator. Blood-stage schizonts were enriched using 45% Percoll (Cytiva, Uppsala, Sweden), centrifuged at 1200 *g* at room temperature. The upper layer was collected, washed twice and resuspended at 0.5% hematocrit in Dulbecco’s phosphate buffered saline (DPBS) (Lonza, Walkersville, MD). Multiwell slides were spotted with 1 µl of enriched blood-stage schizont preparation, air dried, and fixed with cold acetone. Both sporozoite and blood-stage schizont slides were blocked with 1% BSA in PBS (PBS-BSA) for 30 min at room temperature. Serum, two-fold serially diluted in PBS-BSA, was added to the wells, and the slides were incubated in a humidified chamber for 1 h at room temperature. The slides were washed with PBS, and fluorescein isothiocyanate-labeled goat anti-monkey IgG antibody (Santa Cruz Biotechnology, Dallas, TX) at a dilution of 1:200 was added for 30 min at room temperature. Slides were washed, and mounted in Fluoromount-G (Invitrogen, Carlsbad, CA), and viewed with an Olympus microscope. The IFA titer against sporozoites or blood-stage schizonts was defined as the last dilution at which fluorescence intensity was higher than that of baseline serum.

### Intracellular cytokine staining

T cell responses were evaluated using intracellular cytokine staining (ICS). Cryopreserved peripheral blood mononuclear cells (PBMCs) and immune cells isolated from various tissue compartments were thawed at 37 ºC water bath and transferred to 15 ml tube and added 10 ml of pre-warmed RPMI1640 (Gibco) with 20% FBS and centrifuged at 400 g for 10 min at room temperature. Cells were washed once with 5 ml of RPMI 1640 with 10% FBS and were then resuspended in complete media consisting of RPMI1640 supplemented with Antibiotic/Antimycotic, L-glutamine, sodium pyruvate, MEM Nonessential amino acid, 2-mercaptoethanol and 5% FBS (all from Gibco). Overall, cells exhibited good condition with over 95% viability, as assessed by propidium iodide (Invitrogen, Eugene, OR) staining and analysed using BD FACSCanto with BD FACSDiva software (Additional file 2: Fig. S2).

For ICS assay, each sample was run as a single replicate. Mononuclear immune cells (1 × 10^6^ cells in 200 μl of medium) were stimulated for 16 h with cryopreserved 100,000 *P. cynomolgi* sporozoites that were preselected for low mitogenic activity. Mononuclear immune cells cultured with medium served as the control (background), while mononuclear immune cells stimulated with the superantigen staphylococcal enterotoxin B (SEB) (Sigma-Aldrich), (4 μg/ml) were used as the positive control. All cell cultures were supplemented with 1 μg/ml of anti-CD28 (clone L293, BD Biosciences) and 1 μg/ml of anti-CD49 (clone L25, BD Biosciences). During the last 6 h of sporozoite stimulation, Golgiplug (BD Biosciences) was added to prevent cytokine secretion. Then cells were washed and stained with a panel of antibodies specific for surface markers including monoclonal antibodies specific to CD3 (clone SP34, BD Biosciences), CD4 (clone L200, BD Biosciences), CD8 (clone SK1, BD Biosciences), and TCR γδ (clone B1, BioLegend, San Diego, CA). The stained cells were fixed/permeabilized and intracellular cytokine was stained with monoclonal antibody against IFN-γ (clone B 27, BioLegend) at a dilution of 1:60. This concentration was predetermined and found to have low non-specific binding while maximizing specific staining of IFN-γ-producing cells. Finally, the frequency of IFN-γ-secreting T cells were analysed using 6-color flow cytometry (BD FACSCanto) using BD FACSDiva software. Gating strategy for flow-cytometry analysis was shown in Additional file 3: Fig. S3. The samples considered positive were those in which the percentage of cytokine-staining cells was at least twice that for the background [20]. The values of unstimulated controls (background) were deducted from sporozoite-specific T cell responses before being reported.

### Statistical analysis

The data were analysed using SPSS 12.0 for Windows (SPSS Inc., Chicago, IL).

Prior to testing for differences, the antibody titers were subjected to a logarithmic transformation. The differences in antibody and T cell responses were assessed using the Friedman test and the Wilcoxon Signed Rank test (for non-normally distributed data), applying the Bonferroni correction for multiple comparisons. P values of < 0.05 were considered statistically significant.

## Results

### Selecting a sporozoite challenge dose to induce primary infection and subsequent two relapses

High sporozoite challenge dose may overwhelm vaccine-mediated protective immunity. To determine an appropriate sporozoite challenge dose for inducing primary infection and subsequent relapses, different doses (10^5^, 10^4^, and 10^3^ sporozoites) were tested in comparison to the gold standard dose of 10^6^ sporozoites, which is commonly used for anti-hypnozoite drug testing [11].

**Fig. 2:**
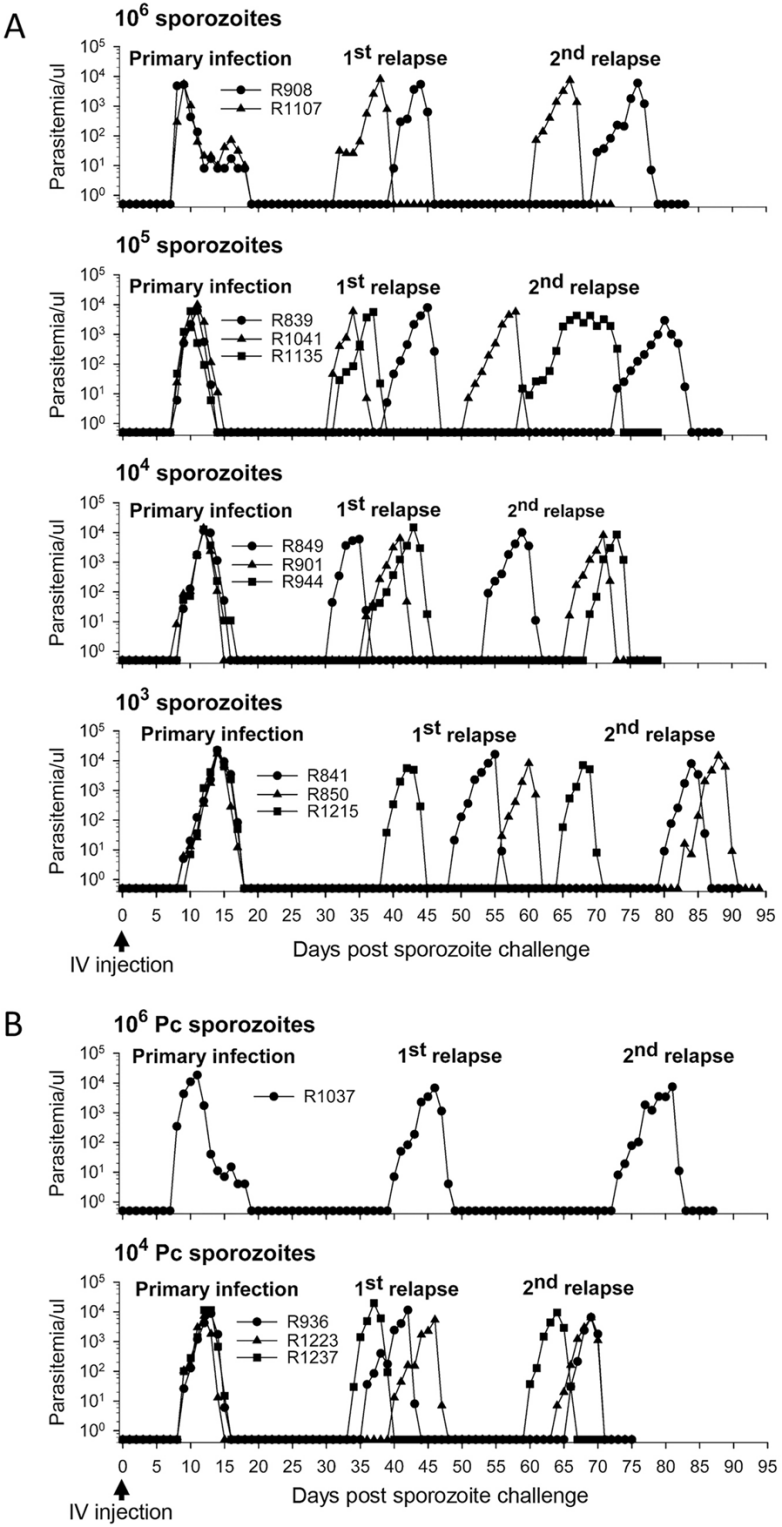
Determination of an appropriate challenge dose of *P. cynomolgi* sporozoites. Animals were IV challenged with varying doses of sporozoites (10^6^, 10^5^, 10^4^, and 10^3^) in PBS containing 5% BSA. The time taken for primary infection and the interval between primary infection and relapses were recorded. A dose of 10^6^ sporozoites was used as a positive control for comparison. Parasitaemia was determined using both thick blood smears (low parasitaemia) and thin blood smears (high parasitaemia). Each parasitaemia episode was treated with CQ and for the last relapse with both CQ and PQ (**A**). A challenge dose of 10^4^ sporozoites was chosen based on the results of the first experiment and was then retested in a second experiment (**B**)

Confirming previous findings [11], it was observed that two animals (R908 and R1107) developed parasitaemia on day 8 after receiving the high dose of 10^6^ sporozoites (Fig. 2A). These animals experienced their first relapse on day 40 and day 32, respectively, and their second relapse on day 70 and day 61, respectively. Animals that received lower challenge doses of 10^5^ sporozoites (R839, R1041, and R1135) and 10^4^ sporozoites (R849, R901, and R944) developed primary infection on day 8 and day 8.7 ± 0.3 (mean ± SE), respectively. The first relapse occurred on day 34 ± 2.5 and day 34.7 ± 1.9 and the second relapse occurred on days 61 ± 6.4 and day 63 ± 4.6, respectively. Animals that received the lowest dose of 10^3^ sporozoites (R841, R850, and R1215) exhibited a primary infection on day 9.3 ± 0.3. The interval between primary infection and relapses increased, with the first relapse occurring on day 48 ± 4.9 and the second relapse on day 76 ± 5.6.

Subsequently, the experiment was repeated with a dose of 10^4^ sporozoites (R936, R1223, and R1237), which produced similar infection times and relapse patterns as before. Specifically, the primary infection occurred on day 9, while the first and second relapses occurred on day 36.7 ± 1.8 and day 63.3 ± 1.8, respectively (Fig. 2B). These findings suggest that a 10^4^ sporozoite dose is a reliable challenge dose to be used in a CPS vaccination study.

### CPS Immunization with two drug regimens

After the initial immunization, all animals (6/6) in group 1 experienced temporary microscopic blood-stage parasitaemia (Additional file 1: Fig. S1). Notably, one animal R1140 continued to have parasitaemia after a 7-day course of AS treatment, necessitating a radical cure consisting of a 7-day treatment of CQ plus PQ for all animals in this group. For the second and third immunizations, AS treatment was replaced with a 7-day CQ treatment followed by a radical cure of CQ plus PQ treatment for another 7 days to ensure blood stage parasite eradication and hypnozoite elimination. Throughout the CPS immunization, no blood parasitaemia was detected in group 2 animals, indicating that a 10-day AP treatment is highly effective in preventing patent blood stage parasite infection. No blood parasitaemia was detected in control animals from group 3 following each immunization.

### Generation of immune responses following CPS immunization

**Fig. 3:**
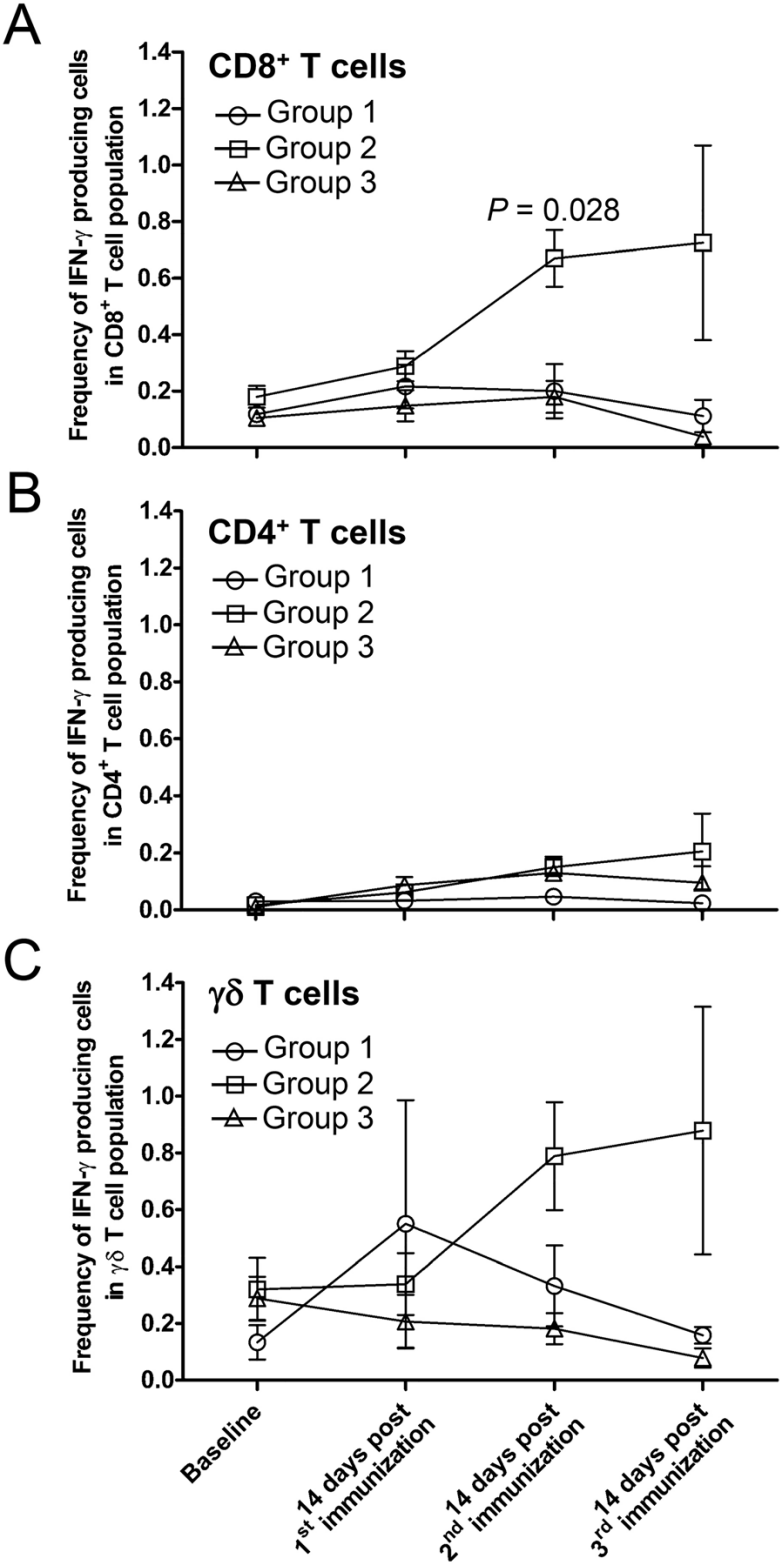
Kinetics of sporozoite-specific peripheral blood IFN-γ T cell responses. PBMC from immunized animals (groups 1 and 2) and control animals (group 3) were evaluated for the sporozoite-specific T cell responses at different time points using an ICS assay: (**A**) CD8^+^, (**B**) CD4^+^ and (**C**) γδ T cells. The data presented in the figure displays the mean ± SE of IFN-γ-producing cells in the CD8^+^, CD4^+^ or γδ T cell population. Individual animal data are provided in Additional file 5: Table S1

This study primarily focused on adaptive T cell responses. Sporozoite-specific peripheral blood T cell responses were measured at baseline and two weeks after each CPS immunization by intracellular IFN-γ staining. Following the second immunization, the frequency of IFN-γ-producing CD8^+^ T cells in the peripheral blood was significantly greater in group 2 animals (mean ± SE = 0.78 ± 0.19) than in group 1 animals (mean ± SE = 0.20 ± 0.09) (Fig. 3A). Sporozoite-specific peripheral blood CD4^+^ T cell responses in both group 1 and group 2 animals were modest with a mean frequency of IFN-γ-producing CD4^+^ T cells ranging from 0.06—0.21%, and there was no significant difference detected between the two groups (Fig. 3B). The study also assessed the immune responses generated by γδ T cells, which are known to be involved in protecting against liver-stage infection [21, 22].

**Fig. 4:**
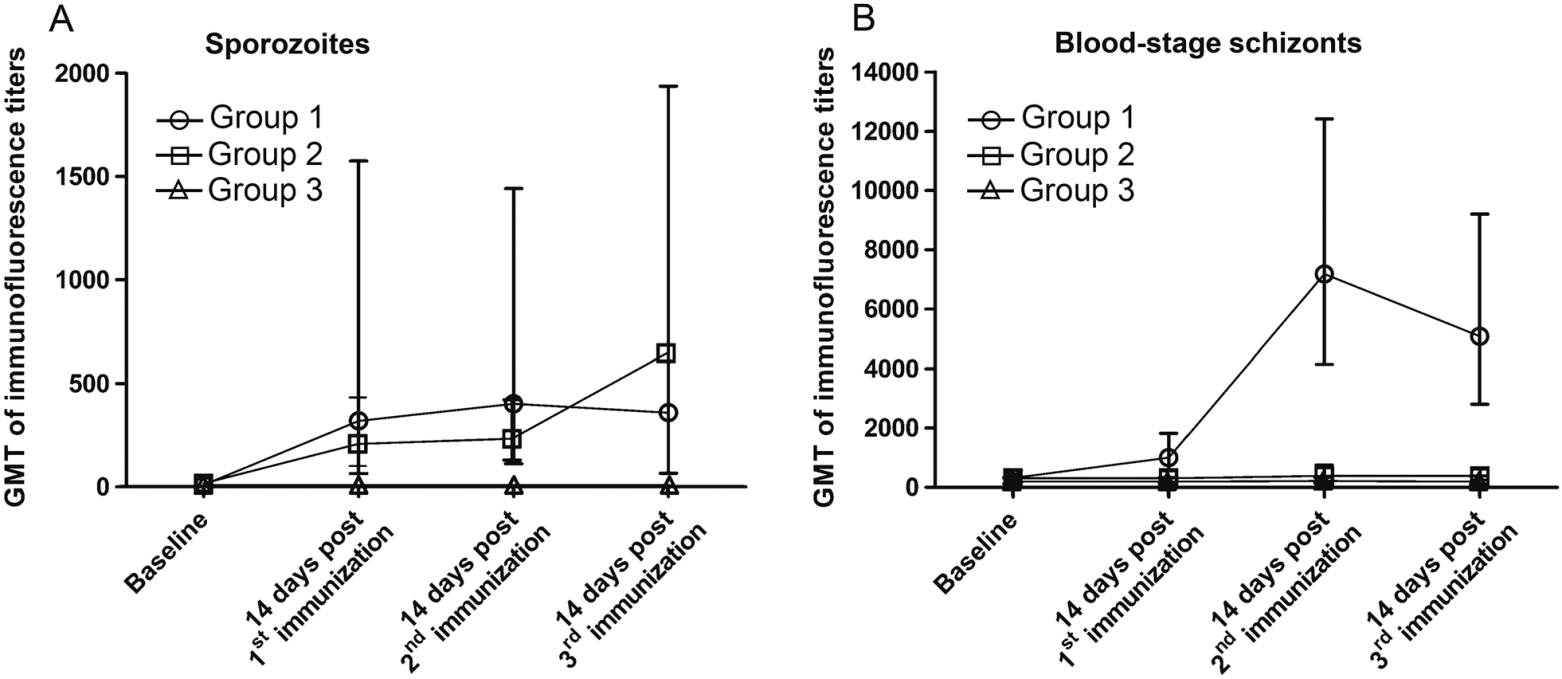
Kinetics of serum antibody responses. Serum samples from immunized animals (group 1 and 2) and control animals (group 3) were measured for antibody responses at different time points using IFA for both *P. cynomolgi* sporozoites (**A**) and *P. cynomolgi* blood-stage schizonts (**B**). Each data point in the graph represents the GMT with 95% CI. Individual animal data are provided in Additional file 6: Table S2

Stimulation of γδ T cells with whole sporozoites in vitro resulted in IFN-γ production. As depicted in Fig. 3C, group 2 animals exhibited higher γδ T cell responses compared to group 1; however, the difference did not reach statistical significance. Negligible CD8^+^, CD4^+^ and γδ T cell responses were observed in the control animals from group 3. To evaluate the antibody responses elicited by CPS immunization, whole sporozoites and blood-stage schizonts were utilized. As demonstrated in Fig. 4A, there was no significant difference in IFA antibody responses to whole sporozoites between the two immunized groups (GMT IFA titers ranging from 65–640). However, animals in group 1 developed IFA antibody responses against blood-stage schizonts, unlike group 2 (Fig. 4B). This finding aligns with the observation that only group 1 animals were exposed to transient blood stage parasites during the CPS immunization course (Additional file 1: Fig. S1). No antibody response to whole sporozoites or blood-stage schizonts was detected in the control animals (group 3).

**Fig. 5:**
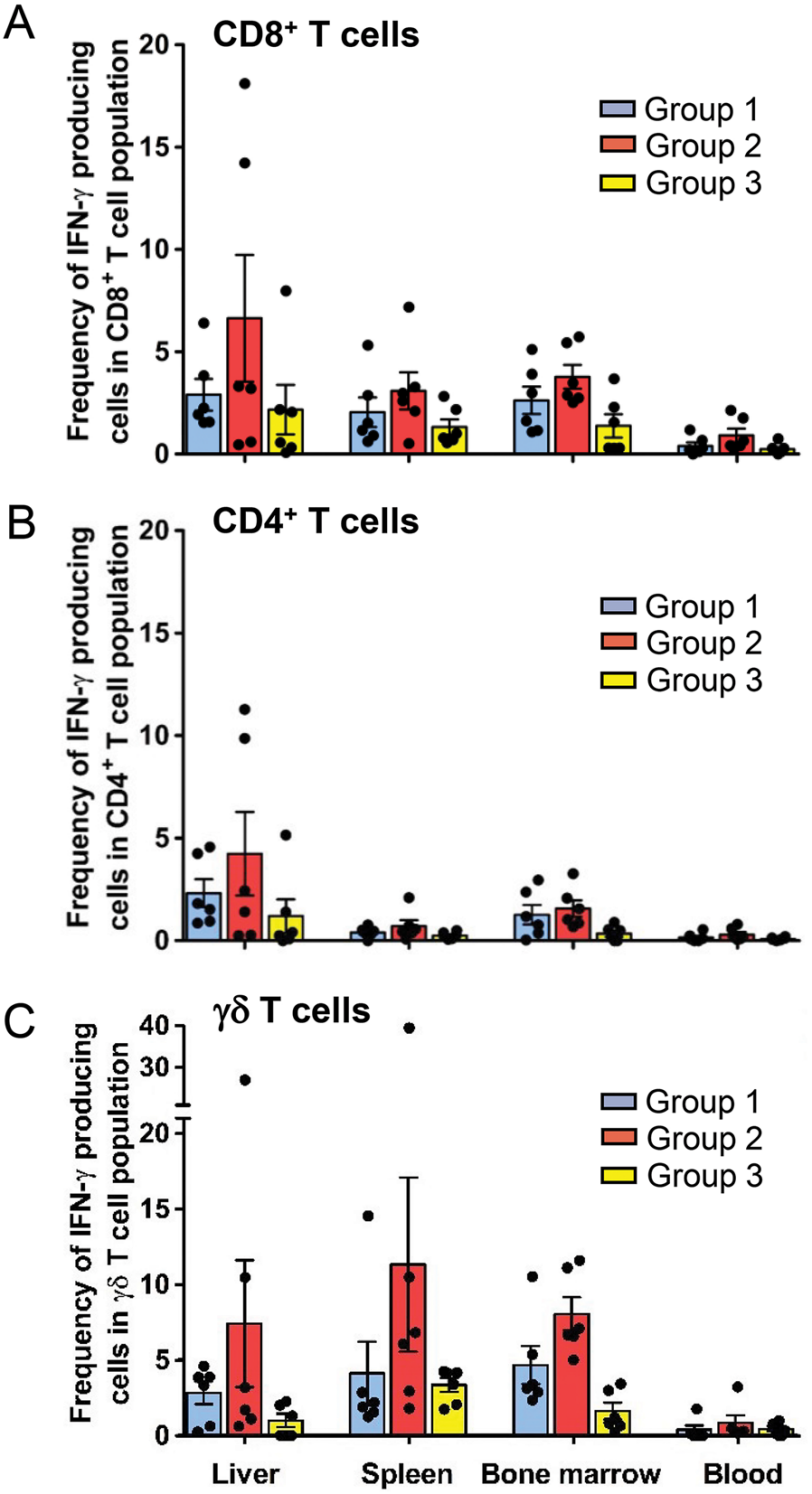
Post-challenge sporozoite-specific T cell responses in various tissue compartments. The frequencies of sporozoite-specific IFN-γ-producing T cells: **(A**) CD8^+^, (**B**) CD4^+^, and (**C**) γδ T cells in the liver, spleen, bone marrow, and peripheral blood were analysed by ICS assay. The data shown are means ± SE. Individual animal data are provided in Additional file 7: Table S3

Assessing T cell responses in peripheral blood only may not reflect protective immunity; therefore, the analysis of T cell responses in different tissue compartments was conducted. All animals were euthanized after challenge at days 194–218, and T cell responses were assessed in the liver, spleen, bone marrow, and peripheral blood (Fig. 5). In the liver, group 2 animals exhibited high levels of sporozoite- specific T cell responses, with a mean frequency ± SE of IFN-γ^+^ CD8^+^ T cells at 6.65 ± 3.09 and a mean frequency ± SE of IFN-γ^+^ CD4^+^ T cells at 4.25 ± 2.03. In the spleen of group 2 animals, the mean frequency ± SE of IFN-γ^+^ CD8^+^ T cells was 3.11 ± 0.91, and the mean frequency ± SE of IFN-γ^+^ CD4^+^ T cells was 0.72 ± 0.29. Additionally, in the bone marrow of group 2 animals, the mean frequency ± SE of IFN-γ^+^ CD8^+^ T cells was 3.79 ± 0.58, and the mean frequency ± SE of IFN-γ^+^ CD4^+^ T cells was 1.58 ± 0.39. However, these differences were not statistically different when compared to group 1 animals (Fig. 5A, B). A high γδ T cell response for group 2 was observed in the spleen (mean frequency ± SE = 11.27 ± 5.77), bone marrow (mean frequency ± SE = 8.00 ± 1.09), and liver (mean frequency ± SE = 7.36 ± 4.20) (Fig. 5C). Nevertheless, the differences were not statistically significant when compared to group 1. Animals in group 1 and group 2 exhibited negligible to modest sporozoite-specific CD8^+^, CD4^+^, and γδ T cell responses in peripheral blood. It should be noted that moderate sporozoite-specific CD8^+^,CD4^+^, and γδ T cell responses were detected in the liver, spleen, and bone marrow of control animals in group 3 at post-challenge suggesting that the observed T cell responses were generated by the challenge sporozoites (on day 112) and not by the CPS immunization.

### Protection outcome after sporozoite challenge

**Fig. 6:**
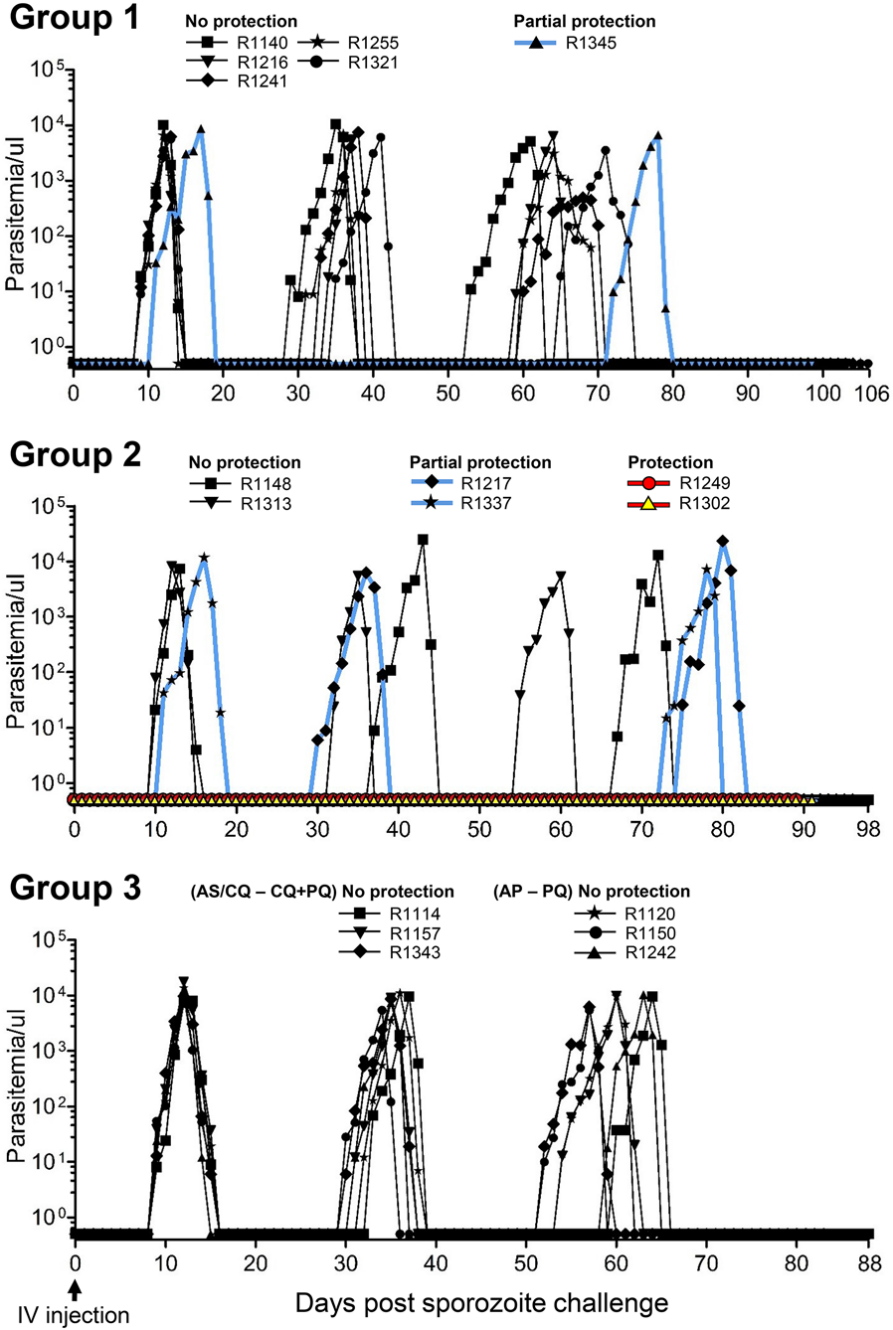
Protection induced by CPS immunization. Following challenge with IV administration of 10^4^ sporozoites on day 112, *P. cynomolgi* blood-stage parasitaemia was measured in all animals until days 82–106 after the challenge using light microscopy. Each episode of parasitaemia (~ 5,000/µl) was treated with CQ for 7 days. The red lines in the graph represent parasitaemia in protected animals and the blue lines represent parasitaemia in partially protected animals

Following the last CPS immunization, all animals received an intravenous challenge with 10^4^ sporozoites on day 112 and were then monitored for the primary infection and subsequent relapses until days 194–218. As shown in Fig. 6, all control animals in group 3 (6/6) developed primary infection on day 9 after challenge, first relapse on day 31.2 ± 0.5, and second relapse on day 55.2 ± 1.4, confirming a successful sporozoite challenge. Similarly, on day 9, five out of six animals in group 1 tested positive for primary infection and had their first and second relapses on day 32.4 ± 1.1 and day 61.5 ± 2.6, respectively. One animal in this group, R1345, demonstrated partial protection as it developed a primary infection on day 11 and experienced a significant delay in relapse, which occurred on day 72. In contrast, four out of six animals in group 2 demonstrated a higher degree of protection than group 1 animals. Two animals, R1249 and R1302, were protected, as they did not have any parasitaemia during primary infection, and subsequent relapses. The other two animals had partial protection; animal R1217 exhibited a significant delay in the onset of first parasitaemia, which was detected on day 30, and a relapse on day 75. Animal R1337 tested positive for primary infection on day 11 and had a marked delay in relapse, which occurred on day 73. Two unprotected animals in this group (R1148 and R1313) developed primary infection on day 10, with their first relapse on day 37 and day 32, respectively, and the second relapse on day 67 and day 55, respectively.

### Potential association between T cell immune response at pre-challenge and post-challenge and protection

**Fig. 7:**
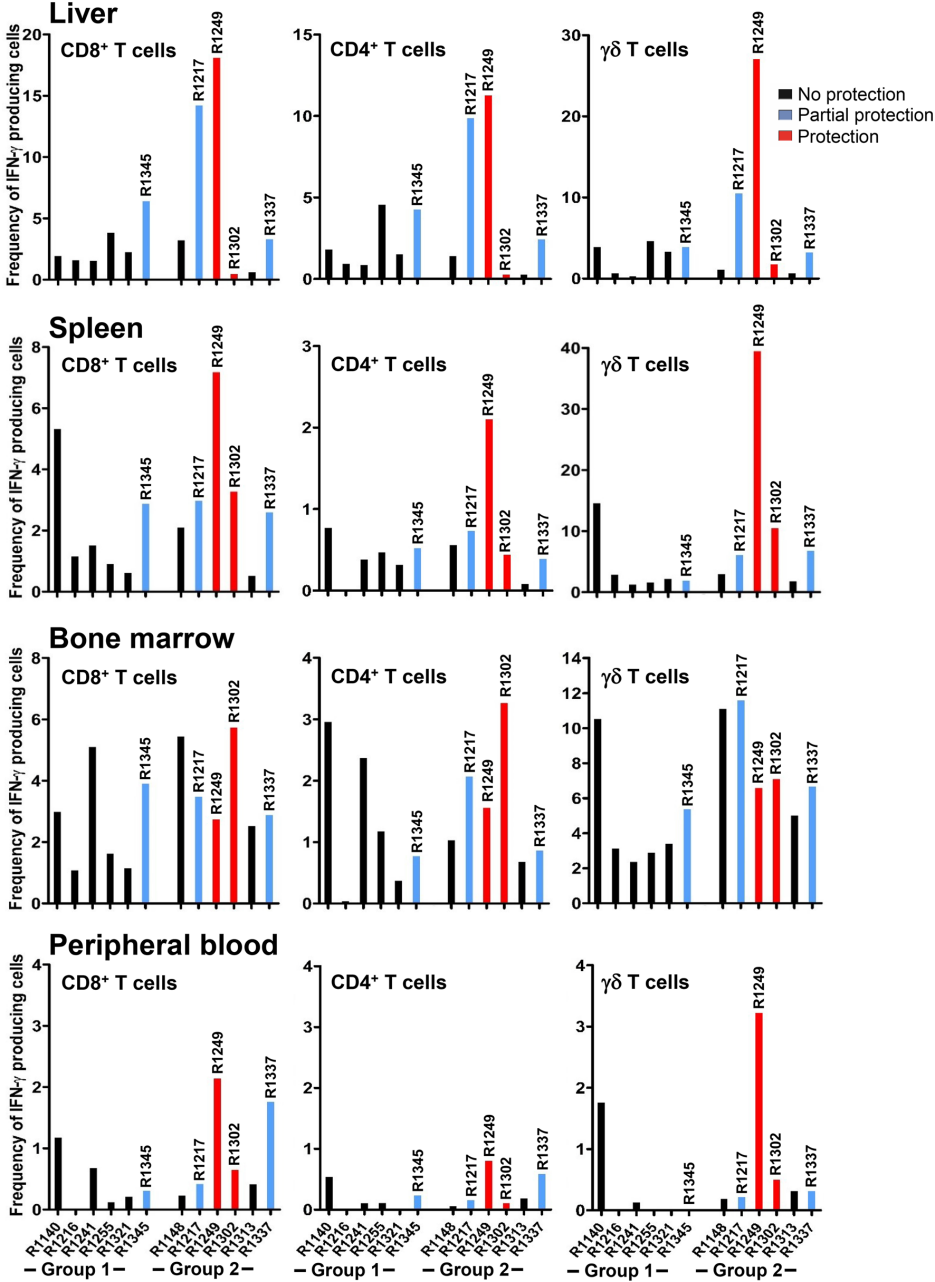
Potential association between T cell immune response in various tissue compartments and protection. Each bar represents the frequency of IFN-γ-producing CD8^+^, CD4^+^ and γδ T cells for each individual animal after challenge (days 194–218)

Given that four animals in group 2 and one animal in group 1 demonstrated different degrees of protection, efforts were then made to identify correlates of T cell immune response. Analyzing the sporozoite-specific response of CD8^+^, CD4^+^ and γδ T cells in peripheral blood at pre-challenge (day 110) (Additional file 4: Fig. S4) and in different tissue compartments at post-challenge (days 194–218) (Fig. 7) did not yield any correlation between protected, partially protected, and unprotected animals. However, one noteworthy finding was that the protected animal (R1249) in group 2 had the highest frequency of IFN-γ^+^ CD8^+^, IFN-γ^+^ CD4^+^, and IFN-γ^+^ γδ T cells in the liver, which were 18.1%, 11.28%, and 27.02%, respectively (Fig. 7). Partially protected animals (R1217 and R1337 in group 2, and R1345 in group (1) showed a relatively high frequency of sporozoite-specific IFN-γ^+^ CD8^+^ (with a mean frequency ranging from 3.3 to 14.23%), IFN-γ^+^ CD4^+^ (with a mean frequency ranging from 2.44 to 9.86%), and IFN-γ^+^ γδ T cell responses (with a mean frequency ranging from 3.19 to 10.47%) in the liver. However, animal R1302 in group 2, which demonstrated protection, had a very low frequency of sporozoite-specific IFN-γ^+^ CD8^+^, IFN-γ^+^ CD4^+^, and IFN-γ^+^ γδ T cells in the liver (0.46%, 0.25%, and 1.72%, respectively), but had moderate to high frequency of sporozoite-specific IFN-γ^+^ CD8^+^, IFN-γ^+^ CD4^+^, and IFN-γ^+^ γδ T cells in spleen (3.28%, 0.44% and 10.49%, respectively) and bone marrow (5.73%, 3.27% and 7.12%, respectively).

## Discussion

This study is the first to demonstrate that CPS immunization under AP – PQ can result in protective immunity in *P. cynomolgi* rhesus macaques, which are an animal model for relapsing vivax malaria. Among the six immunized animals in this group, two developed protection without primary infection and subsequent relapse, and another two showed partial protection.

*Plasmodium cynomolgi* is a nonhuman primate malaria parasite that is closely related to *P. vivax* in terms of both phylogeny and phenotype, including the presence of relapses. The established sporozoite dose for inducing primary infection and relapse in anti-hypnozoite drug screening is 10^6^ sporozoites, which reliably produces 100% infection and a predictable relapse pattern [11]. However, this dose of sporozoites is likely to overcome vaccine-induced protective immunity. The findings demonstrate that IV inoculation with a low dose of 10^4^ *P. cynomolgi* sporozoites consistently induces primary infection and subsequent two relapses, similar to the current gold standard of 10^6^ sporozoites [11].

The use of a relapsing *P. cynomolgi* rhesus macaque model has gained popularity in assessing the efficacy of vaccines in eliciting immune protection. Recent studies have utilized vaccination with PcTRAP and PcCSP through the ChAdOx1 or MVA vaccine platforms, which resulted in good antigen-specific antibody and T cell responses. However, no protection against primary infection or relapses was observed after sporozoite challenge [23]. In a more recent study, the same relapsing *P. cynomolgi* rhesus macaque model was utilized to evaluate the efficacy of CPS immunization. The animals received an IV vaccination with 10^6^ *P. cynomolgi* infectious sporozoites and were allowed to develop primary infection and 3 relapses. Chloroquine was provided after parasitaemia was detected until it was cleared. The results showed that this approach yielded sterile protection in 3 out of 4 immunized animals [24]. However, it should be noted that the animals were challenged with 200 sporozoites, and it is unknown if this low dose could induce a relapse. In addition, sporozoite-specific T cell responses were not investigated.

The CPS immunization in this study employed two drug regimens: AS/CQ, which eliminates blood-stage parasites, and AP, which eliminates blood-stage parasites and liver stage schizonts. To prevent relapse during the course of immunization, a radical cure with CQ plus PQ (group 1) or PQ alone (group 2) was used to eliminate hypnozoites. Interestingly, two-thirds of immunized animals receiving the AP − PQ regimen developed protection (2 animals) and partial protection (2 animals) and developed high CD8^+^, CD4^+^ and γδ T cell responses after the sporozoite challenge. It was surprising that animals that received the AS/CQ − CQ + PQ regimen in this study showed poor protection against sporozoite challenge, although previous animal and human studies have consistently demonstrated that CPS immunization under either AS or CQ drug regimen induces strong T cell responses and high protection [12–15]. Further study is needed to confirm whether the use of AS during the first CPS immunization and CQ during the second and third, as employed in this study, contributes to modest protection.

Animals receiving the AP – PQ regimen during CPS immunization did not experience exposure to blood-stage parasites and, consequently, exhibited no antibody response to blood-stage schizonts. This suggests that immunity to blood-stage parasites may not play a significant role in the protection observed in this animal group. The reason why some animals in the study, that were partially protected, developed a significant delay in the first parasitaemia and a long interval before relapse (R1217) or had a normal primary infection with a long interval before relapse (R1345 and R1337), remains unclear. The findings suggest that the immune protection against the primary infection and relapse is complex and requires further investigation.

Despite both protection and partial protection were observed in this study, specific immune markers directly linked to protection could not be identified. It is important to acknowledge that the small sample size may have hindered the ability to detect immune correlations. Previous studies on CPS immunization have shown that CD8^+^ T cell response in the liver is critical for protective immunity [25, 26]. In this study, one animal that was protected developed very high T cell responses (CD8^+^, CD4^+^, and γδ T cells) in the liver after the challenge, while the partially protected animals also exhibited relatively high T cell responses in the liver. However, there was one animal (R1302) that stood out as an exception. Despite being protected, R1302 exhibited very poor T cell responses in the liver, while maintaining average to high T cell responses in the spleen and bone marrow. The sporozoite-specific T cell response in the liver of monkey R1302 was measured twice, and in both instances, consistent results were obtained (Additional file 7: Table S3). It is speculated that an effective liver innate immune response is responsible for protection in this particular animal and subsequently suppresses the formation of local sporozoite-specific T cell responses in the liver. This hypothesis is supported by recent observations; (1) innate immune responses involving type I IFN signaling in infected liver cells can control liver-stage infection [17]; (2) type I IFN signaling in the liver can dampen the response of antigen-specific CD8^+^ T cells in the liver [27]; and (3) stimulation of innate immune response can confer protection against liver-stage infection but reduces priming of antigen-specific CD8^+^ T cells in mice immunized with radiation-attenuated sporozoites [28].This hypothesis opens avenues for further research into the intricate interplay between innate and adaptive immunity in the context of malaria liver-stage infection.

It is important to acknowledge certain limitations of the study. Firstly, the number of animals in each group was relatively small, which may affect the generalizability of the findings. Secondly, only one protected animal, R1302, exhibited a very poor T cell response in the liver, suggesting the necessity for further replication of this observation. Thirdly, the detection of blood-stage parasitaemia ended at 82–106 days after sporozoite challenge, potentially missing late relapses. Fourthly, parasitaemia was measured by thick blood smear reading, which is not highly sensitive to exclude the possibility that sub-patent parasitaemia was present in the animals who did not develop patent parasitaemias. Additionally, T cell responses in the liver, spleen, or bone marrow were not evaluated before the challenge, which prevents us from establishing a baseline and fully understanding the dynamics of T cell responses in these organs. The T cell responses observed in the immunized groups after the challenge may have been influenced by the challenge sporozoites acting on memory T cells. Therefore, it is crucial that future studies validate the findings to ensure their accuracy and further explore the mechanisms involved.

## Conclusions

This study highlights that CPS immunization with the AP − PQ regimen can generate a robust T cell immune response that offers protection against initial infection and subsequent relapses. It also raises the possibility that an effective liver innate immune response in some animals can control liver-stage infection without the help from adaptive T cell immunity in the liver. The findings emphasize the promising prospects of utilizing relapsing *P. cynomolgi*-infected rhesus monkeys as a model for studying the role of local liver adaptive T cells and the innate immune response in protecting against initial infection and relapse. Furthermore, this model has the potential to guide the development of potent vaccines against relapsing *P. vivax*.

### Supplementary Information

**Additional file 1: Fig. S1.** Transient *P*. *cynomolgi* blood-stage parasitaemia during three CPS immunizations

**Additional file 2: Fig. S2.** Gating strategy for determining the cell viability of mononuclear immune cells before cell culture

**Additional file 3: Fig. S3.** Gating strategy for flow cytometry analysis

**Additional file 4: Fig. S4.** Pre-challenge T cell responses in peripheral blood as possible immune markers for protection

**Additional file 5: Table S1.** Kinetics of sporozoite-specific peripheral blood IFN-γ T cell responses

**Additional file 6: Table S2.** Kinetics of serum antibody responses (IFA)

**Additional file 7: Table S3.** Post-challenge sporozoite-specific T cell responses in various tissue compartments

## Data Availability

The datasets used and/or analysed during the current study are available from the corresponding author on reasonable request.

## Abbreviations

CPS: Chemoprophylaxis with sporozoite
AS: Artesunate
CQ: Chloroquine
PQ: Primaquine
AP: Atovaquone-proquanil
IFN: Interferon
G6PD: Glucose-6-phosphate dehydrogenase
VMP001: Vivax malaria protein 1
IFA: Immunofluorescence assay
ICS: Intracellular cytokine staining
CSP: Circumsporozoite protein
ChAdOx1: Chimpanzee adenovirus
MVA: Modified vaccinia ankara
