# Supplementary material for: Superior protection in a relapsing Plasmodium cynomolgi rhesus macaque model by a chemoprophylaxis with sporozoite immunization regimen with atovaquone-proguanil followed by primaquine
Source: Malar J. 2024 Apr 17;23:106. doi: 10.1186/s12936-024-04933-y (PMC11022453; doi:10.1186/s12936-024-04933-y)
Supplement: Supplementary file 1 — Additional file 1: Fig. S1. Transient P. cynomolgi blood-stage parasitaemia during three CPS immunizations [file 12936_2024_4933_MOESM1_ESM.pdf]

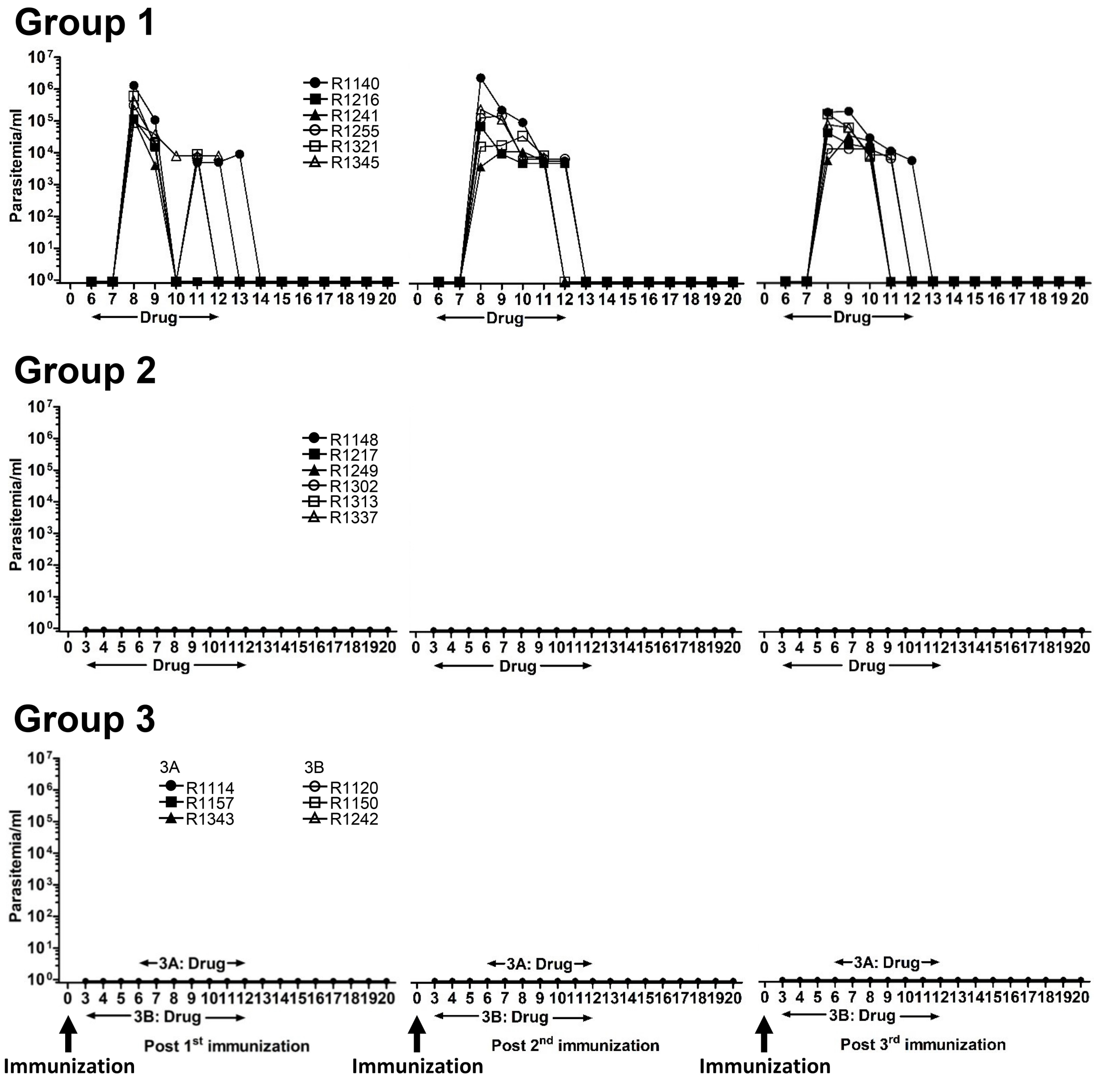

Fig. S1 Transient *P. cynomolgi* blood-stage parasitemia during three CPS immunizations

Eighteen healthy naive rhesus macaques were exposed to 25-35 bites from *P. cynomolgi*-infected mosquitoes (groups 1 and 2) or uninfected mosquitoes (group 3). After each drug treatment during CPS immunization, blood smears were examined daily for parasites until the end of the treatment using thick and thin blood smear microscopy.
