# Supplementary material for: Superior protection in a relapsing Plasmodium cynomolgi rhesus macaque model by a chemoprophylaxis with sporozoite immunization regimen with atovaquone-proguanil followed by primaquine
Source: Malar J. 2024 Apr 17;23:106. doi: 10.1186/s12936-024-04933-y (PMC11022453; doi:10.1186/s12936-024-04933-y)
Supplement: Supplementary file 2 — Additional file 2: Fig. S2. Gating strategy for determining the cell viability of mononuclear immune cells before cell culture [file 12936_2024_4933_MOESM2_ESM.pdf]

Gating strategy for determining the cell viability of mononuclear immune cells before cell culture

1. Add propidium iodide (PI) staining solution to the cell suspension at a final concentration of 2.23 µg/ml.
2. Incubate the cells with PI for 5 minutes in the dark at room temperature.
3. Immediately acquire the stained cells using the FACSCanto and FACSDiva software for data acquisition and analysis
4. Gate mononuclear immune cells based on forward scatter area (FSC-A) versus side scatter area (SSC-A) dot plots, excluding debris.
5. Gate viability and dead cells within mononuclear immune cell gate using FL1-A vs propidium iodide fluorescence base on unstained cells (autofluorescence)

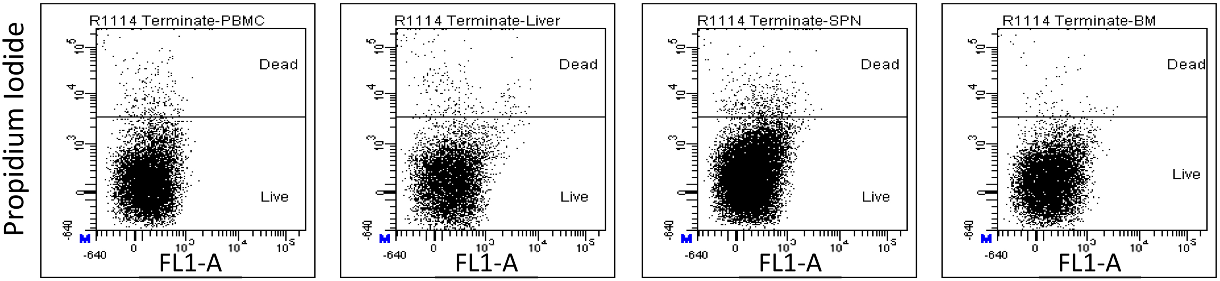

Blood Liver Spleen Bone Marrow
