# Supplementary material for: Superior protection in a relapsing Plasmodium cynomolgi rhesus macaque model by a chemoprophylaxis with sporozoite immunization regimen with atovaquone-proguanil followed by primaquine
Source: Malar J. 2024 Apr 17;23:106. doi: 10.1186/s12936-024-04933-y (PMC11022453; doi:10.1186/s12936-024-04933-y)

Gating strategy for flow cytometry analysis

1. Gate singlet events to exclude cell aggregates using FSC-Area (FSC-A) versus FSC-Height dot plot.
2. Within the singlet population, lymphocytes were gated to exclude cellular debris and dead cells on FSC-A versus side scatter area (SSC-A) dot plot.
3. Within the lymphocyte population, CD3^+^ T cells were gated using CD3 versus SSC-A dot plot.
4. Within the CD3^+^ T cell population, γδ^+^ and γδ^-^ T cells were gated using CD3 versus γδ dot plot.
5. Within the CD3^+^γδ^-^ T cell population, CD4^+^/CD8^-^ and CD4^-^/CD8^+^ populations were gated using CD4 versus CD8 dot plot.
6. Plot a dot plot of IFN-γ versus each T cell subset population.
7. Define gates for IFN-γ^+^ cells based on control tube.

Note: A large number of lymphocytes (~100,000 cells) could be acquired for flow cytometry analysis of each animal, indicating that the cultured mononuclear immune cells for intracellular cytokine staining were likely in good condition.


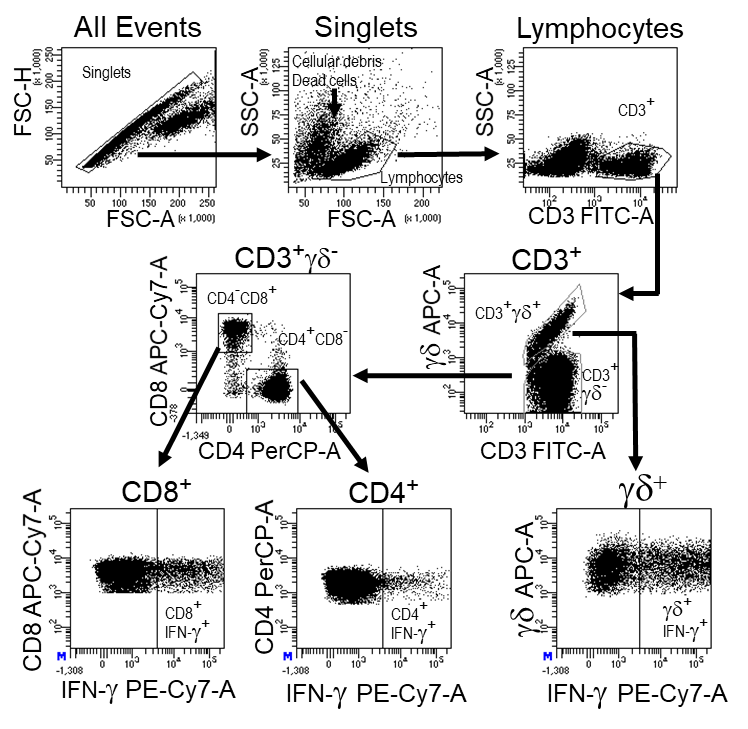

Supplement: Supplementary file 3 — Additional file 3: Fig. S3. Gating strategy for flow cytometry analysis [file 12936_2024_4933_MOESM3_ESM.docx]
