# Supplementary material for: Superior protection in a relapsing Plasmodium cynomolgi rhesus macaque model by a chemoprophylaxis with sporozoite immunization regimen with atovaquone-proguanil followed by primaquine
Source: Malar J. 2024 Apr 17;23:106. doi: 10.1186/s12936-024-04933-y (PMC11022453; doi:10.1186/s12936-024-04933-y)
Supplement: Supplementary file 4 — Additional file 4: Fig. S4. Pre-challenge T cell responses in peripheral blood as possible immune markers for protection [file 12936_2024_4933_MOESM4_ESM.pdf]

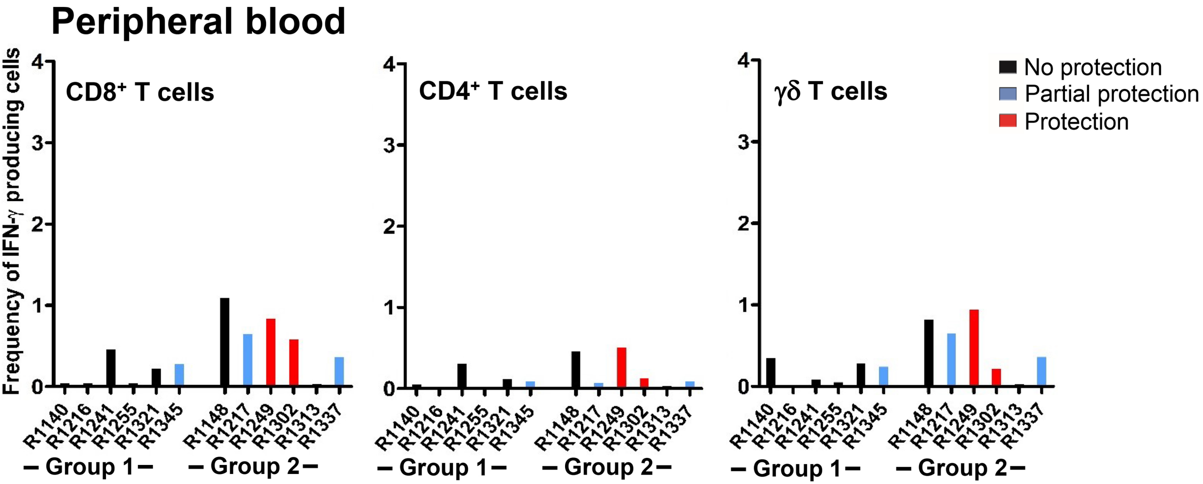

Fig. S4 Pre-challenge T cell responses in peripheral blood as possible immune markers for protection.

Each bar represents the frequency of IFN-γ-producing $CD8^+$, $CD4^+$ and γδ T cells in peripheral blood for each individual animal before challenge (day 110).
